# Decrease of gene expression of astrocytic 5-HT_2B_ receptors parallels development of depressive phenotype in a mouse model of Parkinson’s disease

**DOI:** 10.3389/fncel.2015.00388

**Published:** 2015-10-06

**Authors:** Xique Zhang, Dan Song, Li Gu, Yan Ren, Alexei Verkhratsky, Liang Peng

**Affiliations:** ^1^Department of Neurology, The First Affiliated Hospital, China Medical UniversityShenyang, China; ^2^Laboratory of Brain Metabolic Diseases, Institute of Metabolic Disease Research and Drug Development, China Medical UniversityShenyang, China; ^3^Faculty of Life Science, The University of ManchesterManchester, UK; ^4^Achucarro Center for Neuroscience, IKERBASQUE, Basque Foundation for ScienceBilbao, Spain; ^5^University of Nizhny NovgorodNizhny Novgorod, Russia

**Keywords:** Parkinson’s disease, depression, astrocyte, MPTP model, 5-HT_2B_ receptor, anhedonia

## Abstract

Astrocytes contribute to pathogenesis of neuropsychiatric disorders, including major depression. Stimulation of astroglial 5-HT_2B_ receptors transactivates epidermal growth factor receptors (EGFRs) and regulates gene expression. Previously we reported that expression of 5-HT_2B_ receptors in cortical astrocytes is down-regulated in animals, which developed anhedonia in response to chronic stress; moreover this down-regulation as well as anhedonia, are reversed by chronic treatment with fluoxetine. In this study we have investigated whether astrocytic 5-HT_2B_ receptor is involved in anhedonia in C57BL/6 mice model of Parkinson’ disease (PD) induced by intraperitoneal injection of 1-methyl-4-phenyl-1,2,3,6-tetrahydropyridine (MPTP) for 7 days. The MPTP treatment induced anhendonia in 66.7% of animals. The appearance of depressive behavior was accompanied with motor deficiency and decrease of tyrosine hydroxylase (TH) expression. Expression of mRNA and protein of 5-HT_2B_ receptor in animals that became anhedonic decreased to 77.3 and 79.3% of control groups, respectively; in animals that received MPTP but did not develop anhedonia the expression of 5-HT_2B_ receptor did not change. Experiments with FACS-sorted isolated cells demonstrated that decrease in 5-HT_2B_ receptor expression was confined to astrocytes, and did not occur in neurons. Fluoxetine corrected MPTP-induced decrease of 5-HT_2B_ receptor expression and depressive behavior. Our findings indicate that regulation of gene expression of 5-HT_2B_ receptors in astroglia may be associated with pathophysiological evolution of PD-induced depression.

## Introduction

Parkinson’s disease (PD) is a neurodegenerative disorder originating from a progressive loss of dopamine-containing neurons in the substantia nigra pars compacta (SNpc; Hornykiewicz and Kish, 1987). Clinically, the disease is characterized by resting tremor, slowness of movement, rigidity and postural instability (Fahn and Przedborski, 2000), which are often accompanied with cognitive deficits and behavioral abnormalities. Depression is the most common psychiatric symptom of the PD and is the major factor that negatively impacts on the life quality (Costa et al., 2012). Nevertheless, 5-HT seems to be the most important monoamine related to the pathophysiology and the action of antidepressants. 5-HT deletion may also play roles in motor and non-motor features of PD (Fox et al., 2009).

The morbid changes associated with depression affect not only neurons; there is constantly increasing evidence that astrocytes contribute to pathogenesis of various neuropsychiatric disorders (Verkhratsky et al., 2014). The number of astrocytes is significantly decreased in the brain of depressed patients (McNally et al., 2008), whereas treatment with antidepressants has been shown to increase expression of astrocytic markers glial fibrillary acidic protein (GFAP) and ALDH1L1 (Barley et al., 2009). The role of astroglia in pathogenesis of PD remains to be fully characterized. Importantly, astrocytes specifically express monoamine oxidase-B associated with catabolism of catecholamines and dopamine (Ekblom et al., 1993). Incidentally, increased astrocytic expression of MAO-B in inducible transgenic mouse triggered Parkinsonian symptoms (Siddiqui et al., 2011).

In clinical settings, PD-associated depression is treated with antidepressants, including selective serotonin reuptake inhibitors (SSRIs). Astrocytes express multiple neurotransmitter receptors, that include 5-HT_2_ receptors to serotonin, and α- and β-adrenergic receptors (Li et al., 2008a,b; Du et al., 2010; Ding et al., 2013). Previously, we reported that a SSRI fluoxetine acts as an agonist at 5-HT_2B_ receptors and induces transactivation of epidermal growth factor receptor (EGFR) in astrocytes (Li et al., 2008b). We further discovered that expression of astrocytic, but not neuronal 5-HT_2B_ receptors is down-regulated in cerebral cortex of anhedonic animals, which has been exposed to chronic mild stress (CMS). The down-regulation of 5-HT_2B_ receptors, as well as anhedonia, was reversed by chronic treatment with fluoxetine (Li et al., 2012). This finding suggests the role for astrocytic 5-HT_2B_ receptor in depression.

A “designer drug”, 1-methyl-4-phenyl-1,2,3,6-tetrahydropyridine (MPTP) is most widely used for producing animal models of PD. The MPTP (which by itself is non-toxic), is a lipophilic substance that crosses blood-brain barrier and is accumulated in astrocytes, where MAO-B converts it to the toxic metabolite, MPP+. This latter is released from astrocytes and is transported into dopaminergic neurones by dopamine transporter; subsequently MPP+ acts is a potent inhibitor of mitochondrial Complex I in dopaminergic neurons, thus compromising ATP synthesis, increasing production of reactive oxygen species and leading to cell death (for review, see Meredith et al., 2008). In the present study, we investigate the relationship between gene expression of 5-HT_2B_ receptor and the occurrence of depressive behavior by tests such as sucrose preference test, forced swim test, tail suspension and open field tasks in the MPTP animal model of PD. Motor symptoms and the tyrosine hydroxylase (TH) staining of dopaminergic neurons are also examined in parallel with depressive behavior.

## Materials and Methods

### Animals

Male C57BL/6 mice (Chang Sheng Biotechnology, Benxi, China), weighing 22–26 g, male FVB/NTg(GFAP-GFP)14Mes/J or B6.Cg-Tg(Thy1-YFPH)2Jrs/J mice (The Jackson Laboratory, Bar Harbor, ME, USA), weighing 20–25 g and CD-1 mice (Charles River, Beijing, China), weighing 30–40 g were kept at standard housing conditions with light/dark cycle of 12 h. Water and food were provided *ad libitum*. All experiments were carried out in accordance with the USA National Institute of Health Guide for the Care and Use of Laboratory Animals (NIH Publications No. 8023) and its 1978 revision, and all experimental protocols were approved by the Institutional Animal Care and Use Committee of China Medical University.

### MPTP Treatment

C57BL/6 mice and FVB/NTg(GFAP-GFP)14Mes/J or B6.Cg-Tg(Thy1-YFPH)2Jrs/J mice were daily injected intraperitoneally with MPTP (25 mg/kg dissolved in 0.9% NaCl) or saline for 7 days. C57BL/6 mice were tested: (i) 3 days (*n* = 15); (ii) 7 day (*n* = 25); (iii) 14 day (*n* = 25) or (iv) 21 day after first injection (*n* = 15). FVB/NTg(GFAP-GFP)14Mes/J or B6.Cg-Tg(Thy1-YFPH)2Jrs/J mice were sacrificed 7 day (*n* = 9) or 14 day after first injection (*n* = 9).

### Fluoxetine Treatment

After either 7 days or 14 days of MPTP treatment, mice with anhedonia were daily injected intraperitoneally with fluoxetine (10 mg/kg/d dissolved in 0.9% NaCl) or saline for 14 days.

### Behavioral Testing

After lesioning, C57BL/6 mice were subjected to a series of behavioral tests for motor activity (pole test and rotarod test), depression behavior (sucrose preference test, forced swim, tail suspension and open field tasks).

The pole test is a test for assessing movement disorder in mice, and was performed as previously described (Matsuura et al., 1997) with minor modifications. The mouse was placed head-upward on the top of a vertical rough-surfaced pole (diameter 1 cm; height 55 cm). The time to turn downward from the top (T-turn time) and to descend to the floor (T-LA time) were measured. The total time was recorded with a maximum duration of 30 s.

Rotarod is a test for assessing motor coordination in mice. Mice were placed on a rotating bar set to a rotation speed of up to 18 rpm during the test. The time spent on the rotating bar, known as the latent period, was recorded. Latency to fall was recorded with a stopwatch, with a maximum of 90 s. The test subject was immediately given two trials, and mean latencies were subject to statistical analysis.

Tail suspension test is a despair-based test, measuring the duration of immobility of animals subjected to inexorable conditions. Mice were individually suspended by their tails at the height of 20 cm using a piece of adhesive tape wrapped around the tail 2 cm from the tip. Behavior was videotaped for 6 min. The duration of immobility was measured by an observer blinded to the treatment groups. Mice were considered immobile only when completely motionless, and mice that climbed their tails were excluded from the data (Gorton et al., 2010).

Forced swimming test is also a despair-based test. Mice were dropped individually into glass cylinders (20 × 20 cm) containing 30 cm deep water that maintained at 25 ± 1°C and remained for 6 min. The time of immobility was recorded during the last 4 min of 6-min testing period, followed 2 min of habituation.

Open field test is an anxiety based test. An open field box (60 × 60 × 40 cm) divided in to 9 squares was used. Mice were placed into the center square. Behaviors were videotaped for 5 min. The parameters used for analysis included total travel distance and time spend in the central area.

The sucrose preference test is a reward-based test and performed as a measure of anhedonia, the lack of interest in pleasant activities. Anhedonia is one, but not the only, characteristic symptom of major depression (Li et al., 2012). Baseline sucrose preference was measured before lesion. After 20 h of food and water deprivation, mice were placed in individual cages and presented with two pre-weighted bottles, one containing 2.5% sucrose solution and another filled with water for 2 h. Percent preference was calculated according to the following formula: % preference = [sucrose intake/(sucrose + water intake)]· 100. A decrease of sucrose preference below 65% was taken as the criterion for anhedonia. This criterion was based on the fact that none of the control mice exhibited less than or equal to 65% preference for sucrose at that time point of the experiment.

Sickness behaviors were measured by body weight, line crossing and social behavior according to the methods of Godbout et al. (2005). Mice were maintained in their home cage (26 × 20 cm) which was divided into six identical rectangles, and locomotor activity was video recorded during 3 min tests. A trained observer who was blind to experimental treatments determined the incidence of line crossing. For social exploration, a CD1 juvenile mouse was introduced into the test subject’s home cage for a 5-min period. The time spent by the experimental mouse showing exploratory behavior towards the caged juvenile was recorded by a trained person blind to treatment. Social behavior was determined as the amount of time that the experimental subject spent investigating (e.g., anogenital sniffing, trailing) the juvenile.

### TH Immunohistochemistry

Mice were anesthetized with pentobarbital and perfused with 0.9% saline, followed by 4% paraformaldehyde in 0.1 M PBS. Brains were extracted and post-fixed in 4% paraformaldehyde overnight at 4°C. They were transferred to a 30% sucrose solution. The cryoprotection brains were sectioned serially at 30 μm using a cryostat microtome. Brain slices were pre-incubated in 0.3% Triton X-100 for 30 mins. Then, sections were incubated with mouse anti-TH antibody (1:1000) overnight at 4°C. After re-warming at 37°C for 30 min, biotin labelled goat anti-rabbit serum (the second antibody) and horseradish peroxidise labelled streptavidin were added in turn according to the instruction of UltraSensitive^TM^ SP IHC Kit (Maixin Biotech., Fuzhou, China). The color was shown with 3,3’-diaminobenzidine tertrahydrochloride (DAB). All sections were air-dried, dehydrated in ascending grades of ethanol, cleaned in xylene and fixed on coverslips with mounting media. Images were obtained using a bright-field microscope.

### Preparation of Freshly Isolated Cells

FVB/NTg(GFAP-GFP)14Mes/J or B6.Cg-Tg(Thy1-YFPH)2Jrs/J mice express fluorescent marker under control of a cell-specific promoter (GFAP for astrocytes or Thy1 for neurones) thus allowing fluorescence-activated sorting of specified cell fractions (Lovatt et al., 2007). Fluorescence-activated sorting was performed as described previously (Wang et al., 2015). Immediately after decapitation, cerebral hemispheres (without olfactory bulbs and hippocampi) were removed. The remaining parts of the brains were placed in cold Hanks buffer containing glutamate receptor antagonists (3 μM DNQX and 100 μM APV), cut into small pieces, and digested with 8 U/ml papain in Ca^2+^/Mg^2+^-free PIPES/cysteine buffer, pH 7.4, for 1 h at 37°C in a humidified atmosphere of CO_2_/air (5:95%). After one wash, the tissue was further digested with 40 U/ml DNase I in Mg^2+^-containing minimum essential medium (MEM) with 1% bovine serum albumin (BSA) for 15 min at 37°C in a humidified atmosphere of CO_2_/air (5:95%). It was then carefully triturated in cold MEM with 1% BSA, and centrifuged over a 90% Percoll gradient, followed by collection of all cells below and including the lipid layer. This suspension was further diluted five times, with MEM containing 1% BSA, and centrifuged to collect the pellet. Immediately thereafter, the cells were re-suspended in cold MEM with 1% BSA and 4 μg/ml propidium iodide and sorted by fluorescence-activated cell sorting (FACS) using the BD FACSAria Cell Sorting System (35 psi sheath pressure, FACSDiva software S/W 2.2.1; BD Biosciences, San José, CA, USA) as described by Lovatt et al. (2007). GFP and YFP were excited by a 488 nm laser, and emissions were collected by 530 nm discrimination filters. Cell identity and purity were verified by mRNA expression of cell markers of astrocytes, neurones and oligodendrocytes, analyzed by reverse-transcription polymerase chain reaction (RT-PCR), in astrocytic and neuronal cell preparations. As shown previously (Fu et al., 2012) there is no contamination with neuronal or oligodendrocytic genes in the samples of astrocytes or of astrocytic or oligodendrocytic genes in the neuronal samples. Since only a relatively small amount of cells is obtained, mRNA but not protein (requiring a larger amount of cells) could be determined.

### Primary Cultures of Astrocytes

Primary cultures of mouse astrocytes were prepared from the neopallia of the cerebral hemispheres of newborn CD-1 mice as previously described (Hertz et al., 1978, 1998; Wang et al., 2015), sparsely seeded, and grown in Dulbecco’s Minimum Essential Medium (DMEM) with 7.5 mM glucose. After 2 weeks *in vitro*, 0.25 mM dibutyryl cyclic AMP (dBcAMP) was included in the medium. These cultures are highly enriched in astrocytes (>95% purity of glial fibrillary protein-(GFAP-) and glutamine synthetase-expressing astrocytes (Hertz et al., 1985). Addition of dBcAMP leads to a morphological and functional differentiation as evidenced by the extension of cell processes and increases in several metabolic and functional activities characteristic of astrocytes *in situ* (Meier et al., 1991). MPTP at 20 μM was added to the culture after 3 weeks of culturing and for 8, 12 and 24 h.

### Reverse Transcription-Polymerase Chain Reaction (RT-PCR)

RT-PCR was performed as described previously (Wang et al., 2015). For determination of mRNA expression of 5-HT_2B_ receptor by RT-PCR, all samples from cerebral cortex, freshly isolated cells or astrocyte cultures were homogenized in Trizol. The RNA pellet was precipitated with isopropanol, washed with 70% ethanol, and dissolved in 10 μl sterile, distilled water, and an aliquot was used for determination of the amount of RNA (Kong et al., 2002).

RT was initiated by a 5 min-incubation at 65°C of 1 μg RNA extract with Random Hexamer at a final concentration of 12.5 ng/l and deoxy-ribonucleoside triphosphates (dNTPs) at a final concentration of 0.5 mM. The mixture was rapidly chilled on ice and briefly spun, and 4 μl 5 × First-Strand Buffer, 2 μl 0.1 M dithiotreitol and 1 μl RNase OUT Recombinant RNase Inhibitor (40 U/μl) were added. After the mixture had been incubated at 42°C for 2 min, 1 μl (200 U) of Superscript II was added, and the incubation at 42°C continued for another 50 min. Subsequently the reaction was inactivated by heating to 70°C for 15 min, and the mixture was chilled and briefly centrifuged.

Polymerase chain reaction (PCR) amplification was performed in a Robocycler thermocycler with 0.2 μM of sense or antisense and 0.375 U of Taq polymerase for 5-HT_2B_ receptor (forward; 5′ CTCGGGGGTGAATCCTCTGA 3′; reverse; 5′ CCTGCTCATCACCCTCTCTCA 3′; Kong et al., 2002), for TATA box-binding protein (TBP), used as a housekeeping gene (forward; CCACGGACAACTGCGTTGAT; reverse; GGCTCATAGCTACTGAACTG; el-Marjou et al., 2000). Initially the template was denatured by heating to 94°C for 2 min, followed by 2.5 min amplification cycles, each consisting of two 45-s periods and one 60-speriod, the first at 94°C, the second at 57°C for 5-HT_2B_ receptor, and at 55°C for TBP, and the third at 72°C. The final step was extension at 72°C for 10 min. The PCR products were separated by 1% agarose gel electrophoresis, stained with 0.5 μg/ml ethidium bromide, and captured by Fluorchem 5500 (Alpha Innotech Corporation, San Leandro, CA, USA). The sizes of the PCR product of 5-HT_2B_ receptor was 370 bp and that of TBP 236 bp.

### Western Blotting

Western blotting was performed as described previously (Wang et al., 2015). Protein content was determined by the Lowry method (Lowry et al., 1951), using BSA as the standard. Samples containing 50 μg protein were applied on slab gels of 10% polyacrylamide and electrophoresed. After transfer to polyvinylidene fluoride (PVDF) membranes, the samples were blocked by 5% skim milk powder in TBS-T (30 mM Tris-HCl, 125 mM NaCl, 0.1% Tween 20) for 1 h. The PVDF membranes were incubated with the first antibody, specific to 5-HT_2B_ receptor overnight at 4°C or β-actin for 2 h at room temperature. After washing, the blots were incubated with peroxidase-conjugated affinity-purified goat-anti-rabbit or goat-anti-mouse horseradish antibody for 2 h. Staining was visualized by ECL detection reagents. Digital images obtained using Gel-Imaging System (Tanon 4200, Shanghai, China). Optical density for each band was assessed using the Window AlphaEase TM FC 32-bit software. Ratios were determined between scanned 5-HT_2B_ receptor and β-actin, used for housekeeping.

### Statistics

Differences between multiple groups were evaluated by one-way analysis of variance (ANOVA) followed by Fisher’s least significant difference (LSD) multiple comparison test for unequal replications. Spearman correlation was used to assess associations between gene expression of 5-HT_2B_ receptor and sucrose consumption. The level of significance was set at *p* < 0.05.

### Materials

Most chemicals, including MPTP, BSA, DNase I, propidium iodide, 6,7-dinitroquinoxaline-2,3-dione (DNQX), 2-amino-5-phosphonovalerate (APV) and first antibodies, raised against β-actin were purchased from Sigma (St. Louis, MO, USA). Anti-TH antibody [EP1533Y] are purchased from Abcam (Cambridge, UK) and UltraSensitive^TM^ SP IHC Kit from Maixin Biotech. (Fuzhou, China). BD Biosciences (Franklin Lakes, NJ, USA) supplied the first antibody, raised against 5-HT_2B_ receptor. The second antibody goat anti-mouse IgG HRP conjugate was from Promega (Madison, WI, USA) and goat anti-rabbit IgG HRP conjugate from Santa Cruz Biotechnology (Santa Cruz, CA, USA). ECL detection reagents were from Amersham Biosciences, Buckinghamshire, UK. Random Hexamer, deoxyribonucleotide triphosphates (dNTPs) and Taq-polymerase for RT-PCR were purchased from TaKaRa Biotechnology Co., Ltd. (Dalian, China), and Superscript II from Gibco Life Technology Invitrogen (Grand Island, NY, USA). Chemicals for preparation of culturing medium were purchased from Sigma (St. Louis, MO, USA) and horse serum from Invitrogen (Carlsbad, CA, USA). Flurchem 5500 was from Alpha Innotech (San Leandro, CA, USA).

## Results

### Effects of MPTP on Expression of mRNA and Protein of 5-HT_2B_ Receptor

**Figure 1:**
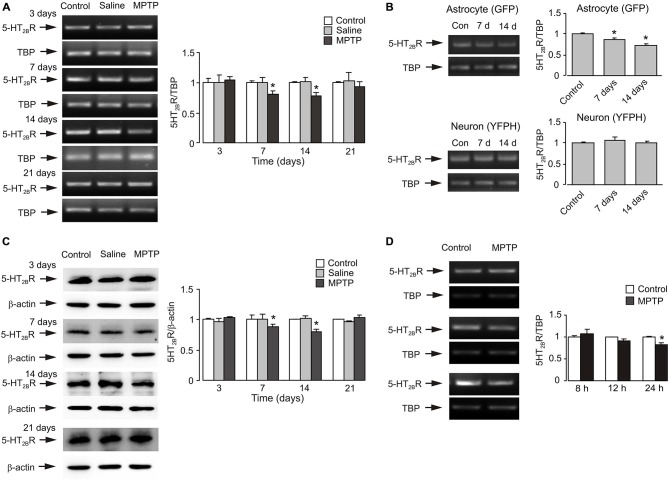
**MPTP induced down-regulation of expression of mRNA and protein of 5-HT_2B_ receptor in brain, in astrocytes isolated by FACS from cerebral hemispheres *in vivo* from mice and in astrocytes in primary cultures. (A)** mRNA expression measured by RT-PCR of 5-HT_2B_ receptor in cerebral cortex* in vivo* from C57BL/6 mice treated with MPTP (25 mg/kg/d, 7 days, i.p.). A representative experiment showing mRNA for 5-HT_2B_ receptor and for TATA BOX-BINDING PROTEIN (TBP), as a housekeeping gene. The size of PCR product of 5-HT_2B_ receptor is 370 bp, and that of TBP 236 bp. Similar results were obtained in six independent experiments. Average mRNA expression was quantified as ratio between 5-HT_2B_ receptor and the housekeeping TBP gene. Ratios between 5-HT_2B_ receptor and TBP in control group were designated a value of one. SEM values are indicated by vertical bars. *Indicates statistically significant (*p* < 0.05) difference from control and saline group at the same treatment period. **(B)** mRNA expression measured by RT-PCR of 5-HT_2B_ receptor in astrocytes and neurons isolated by FACS from cerebral hemispheres *in vivo* from mice (FVB/NTg(GFAP GFP)14Mes/J or B6.Cg-Tg(Thy1-YFPH)2Jrs/J) treated with MPTP (25 mg/kg/d, 7 days, i.p.). A representative experiment showing mRNA for 5-HT_2B_ receptor and for TBP, as a housekeeping gene. Similar results were obtained in three independent experiments. Average mRNA expression was quantified as ratios between 5-HT_2B_ receptor and the housekeeping TBP gene. Ratios between 5-HT_2B_ receptor and TBP in control group were designated a value of one. SEM values are indicated by vertical bars. *Indicates statistically significant (*p* < 0.05) difference from other groups in the same cell type. **(C)** Protein expression measured by immunoblotting of 5-HT_2B_ receptor (55 kDa) in cerebral cortex* in vivo* from C57BL/6 mice treated with MPTP (25 mg/kg/d, 7 days, i.p.). Immunoblots from representative experiments. Bands of 55 and 42 kDa represent 5-HT_2B_ receptor and β-actin, respectively. Similar results were obtained in six independent experiments. Average protein level was quantified as ratios between 5-HT_2B_R and β-actin. Ratios between 5-HT_2B_ receptor and TBP in control group were designated a value of one. SEM values are indicated by vertical bars. *Indicates statistically significant (*p* < 0.05) difference from other groups at the same treatment period. **(D)** mRNA expression of 5-HT_2B_R in primary cultures of astrocytes. Cells were treated with saline (control) or MPTP (20 μM) for 8, 12 and 24 h. Southern blot from a representative experiment. The size of 5-HT_2B_R is 370 bp, and TBP 236 bp. **(B)** Average mRNA expression was quantitated as ratios between 5-HT_2B_R and TBP. S.E.M. values are indicated by vertical bars, *n* = 6, *Indicates statistically significant (*p* < 0.05) difference from other groups at the same treatment period.

The time course of expression of 5-HT_2B_ receptor mRNA during MPTP intraperitoneal injection for 7 days is shown on Figure 1A. At 3 days, there was no difference in 5-HT_2B_ receptor expression in cerebral tissues obtained from control, saline and MPTP groups. However, after 7 days of treatment, mRNA of 5-HT_2B_ receptor decreased to 80.7% of control and saline groups (*n* = 6, *p* < 0.05). It was further decreased to 77.3% after 14 days of treatment (7 days after the cessation of MPTP treatment; *n* = 6, *p* < 0.05). At 21 days (14 days after the end of MPTP treatment), mRNA of 5-HT_2B_ receptor was returned to the control level. Experiments with freshly isolated astrocytes and neurones from transgenic mice demonstrated that the decrease of 5-HT_2B_ receptor mRNA expression in the *in vivo* brain was confined to astrocytes and was not detected in neurones (Figure 1B). Expression of 5-HT_2B_ receptor protein showed similar pattern as mRNA (Figure 1C). Expression of 5-HT_2B_ receptors was reduced in the brains of MPTP-treated mice to 88.2% of control after 7 days and to 79.3% of control after 14 days (7 days after the end of MPTP treatment; *n* = 6, *p* < 0.05), and returned to the control level after 21 days (14 days after the end of MPTP treatment; *n* = 6, *p* < 0.05). Effects of MPTP on gene expression of 5-HT_2B_ receptor in cultured astrocytes is shown on Figure 1D. At 3 and 12 h, there was no difference in 5-HT_2B_ receptor expression between control and MPTP groups. However, after 24 h of exposure, mRNA of 5-HT_2B_ receptor decreased to 81.6% of control group (*n* = 6, *p* < 0.05).

### Effects of MPTP on Depression Behavior

**Figure 2:**
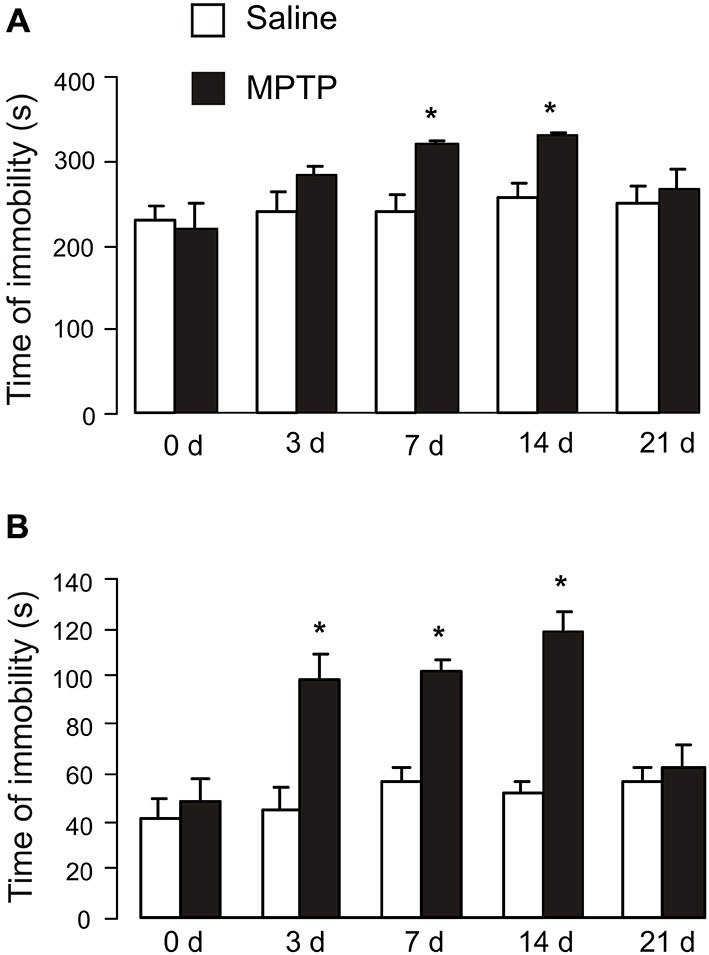
**Effects of MPTP on tail suspension test and forced-swimming test. (A)** Tail suspension test. C57BL/6 mice with or without MPTP (25 mg/kg/d, 7 days, i.p.) were individually suspended from their tails at the height of 20 cm using a piece of adhesive tape wrapped around the tail 2 cm from the tip. Behavior was videotaped for 6 min. The values are expressed as the mean ± SEM, *n* = 6, *Indicates statistically significant (*p* < 0.05) difference from other groups at the same treatment period. **(B)** Forced-swimming test. C57BL/6 mice with or without MPTP treatment (25 mg/kg/d, 7 days, i.p.) were dropped individually into glass cylinders (10 × 10 cm) containing 10 cm deep water that maintained at 25 ± 1°C and remained for 6 min. The time of immobility was recorded during the last 4 min of 6-min testing period, followed 2 min of habituation. The values are expressed as the mean ± SEM, *n* = 5–6, *Indicates statistically significant (*p* < 0.05) difference from other groups at the same treatment period.

The duration of immobility of tail suspension test and time of immobility of forced-swimming test are presented in Figure 2. Exposure to MPTP for 3 days had no effect on duration of immobility (saline: 239.2 ± 23.4 s, *n* = 6; MPTP: 280.8 ± 10.7 s, *n* = 6; *p* > 0.05; Figure 2A). Seven days of MPTP treatment significantly increased the duration of immobility (saline: 236.1 ± 20.8 s, *n* = 6; MPTP: 318.7 ± 4.2 s, *n* = 9; *p* < 0.05), and the duration further increased after 14 days (7 days after the end of MPTP treatment; saline: 253.9 ± 18.2 s, *n* = 6; MPTP: 327.3 ± 3.4 s, *n* = 8; *p* < 0.05). The duration of immobility in MPTP-treated mice then started to decrease and was not significantly different from control group after 21 days (14 days after the end of MPTP treatment; saline: 249.1 ± 17.2 s, *n* = 6; MPTP: 264.1 ± 22.9 s, *n* = 6; *p* > 0.05).

In forced-swimming test, exposure to MPTP for 3 days significantly increased the time of immobility (saline: 45.6 ± 11.2 s, *n* = 5; MPTP: 84.1 ± 9.9 s, *n* = 6; *p* < 0.05; Figure 2B); immobility duration further increased after 7 days (saline: 55.3 ± 9.1 s, *n* = 5; MPTP: 104.5 ± 6.6 s, *n* = 6; *p* < 0.05), and reached maximum after 14 days (7 days after the end of MPTP treatment; saline: 47.0 ± 3.4 s, *n* = 5; MPTP: 135.4 ± 3.9 s, *n* = 6; *p* < 0.05). The time of immobility in MPTP-treated mice returned to control level after 21 days (14 days after the end of MPTP treatment; saline: 60.0 ± 3.3 s, *n* = 5; MPTP: 62.0 ± 13.5 s, *n* = 6; *p* > 0.05).

**Figure 3:**
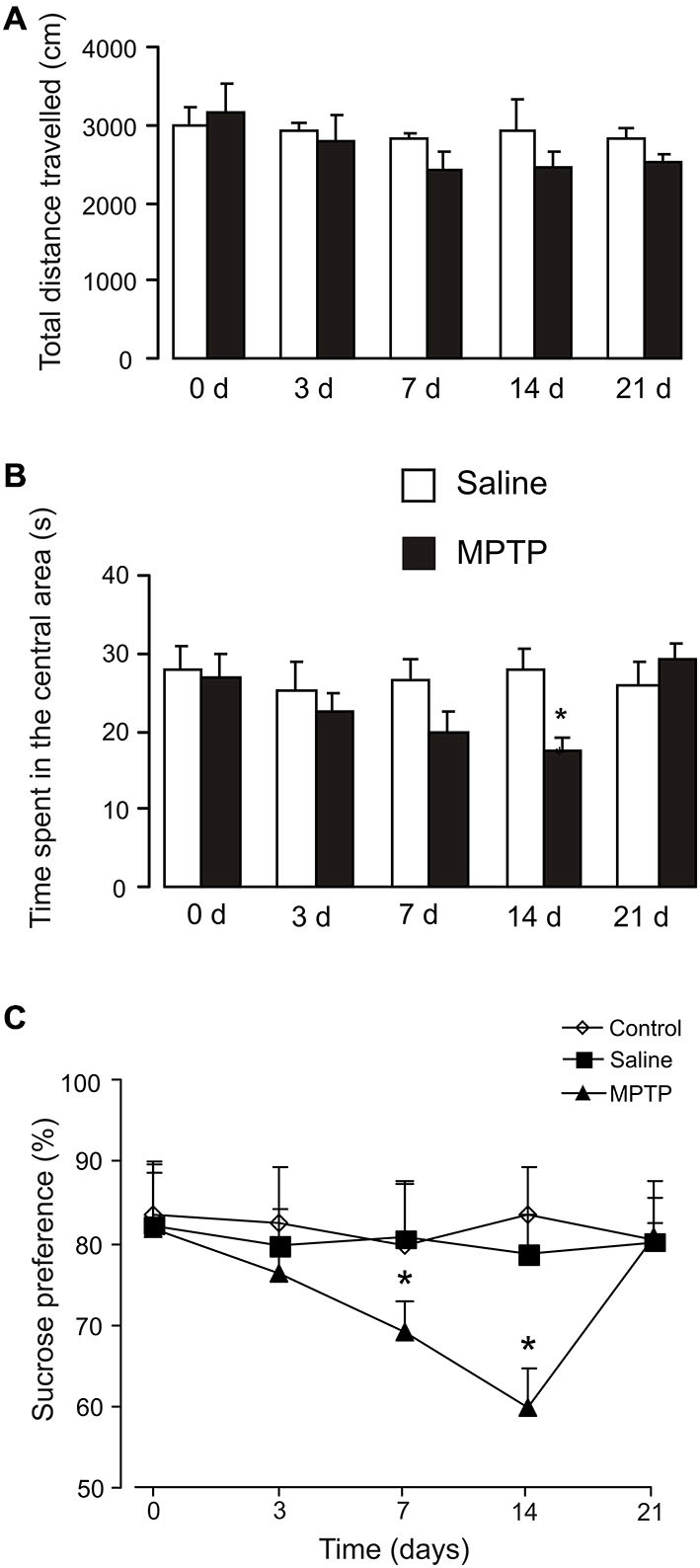
**Effects of MPTP on open field test and sucrose consumption. (A)** The total distance traveled in the central in open field test. C57BL/6 mice with or without MPTP treatment (25 mg/kg/d, 7 days, i.p.) were placed into the center square. Behaviors were videotaped for 5 min. The values are expressed as the mean ± SEM, *n* = 5–7, *Indicates statistically significant (*p* < 0.05) difference from other groups at the same treatment period. **(B)** The time spend in the central in open field test. C57BL/6 mice with or without MPTP treatment (25 mg/kg/d, 7 days, i.p.) were placed into the center square. Behaviors were videotaped for 5 min. The values are expressed as the mean ± SEM, *n* = 5–7, *Indicates statistically significant (*p* < 0.05) difference from other groups at the same treatment period. **(C)** Percentage of sucrose preference measured in C57BL/6 mice with or without MPTP treatment (25 mg/kg/d, 7 days, i.p.). Values were expressed as the mean ± SEM (control: *n* = 6; saline: *n* = 6; MPTP: *n* = 9). *Indicates statistically significant (*p* < 0.05) difference from other groups at the same treatment period.

The total distance traveled and time spend in the central in open field test are presented in Figures 3A,B. Exposure to MPTP for 3 days had no effect on the total distance traveled at all the time points (Figure 3A), but decreased time spend in the central area after 14 days (saline: 27.6 ± 2.9 s, *n* = 5; MPTP: 17.46 ± 1.6 s, *n* = 6; *p* < 0.05; Figure 3B).

Changes in the consumption of sucrose, indicative of MPTP-induced anhedonia are shown on Figure 3C. Exposure to MPTP for 3 days had no effect on sucrose consumption (control: 82.77 ± 6.61%, *n* = 6; saline: 79.62 ± 4.71%, *n* = 6; MPTP: 76.43 ± 3.61%, *n* = 9; *p* > 0.05). Seven days of MPTP treatment decreased sucrose consumption significantly (control: 79.63 ± 8.2%, *n* = 6; saline: 80.65 ± 6.75%, *n* = 6; MPTP: 69.34 ± 3.67%, *n* = 9; *p* < 0.05), and it further decreased after 14 days (7 days after the end of MPTP treatment; control: 83.41 ± 6.14%, *n* = 6; saline: 78.59 ± 5.00%, *n* = 6; MPTP: 59.80 ± 5.00%, *n* = 9; *p* < 0.05). The consumption of sucrose in MPTP-treated mice returned to control level after 21 days (14 days after the end of MPTP treatment; control: 80.39 ± 5.18%, *n* = 6; saline: 80.24 ± 2.37%, *n* = 6; MPTP: 81.05 ± 6.79%, *n* = 9; *p* > 0.05).

**Figure 4:**
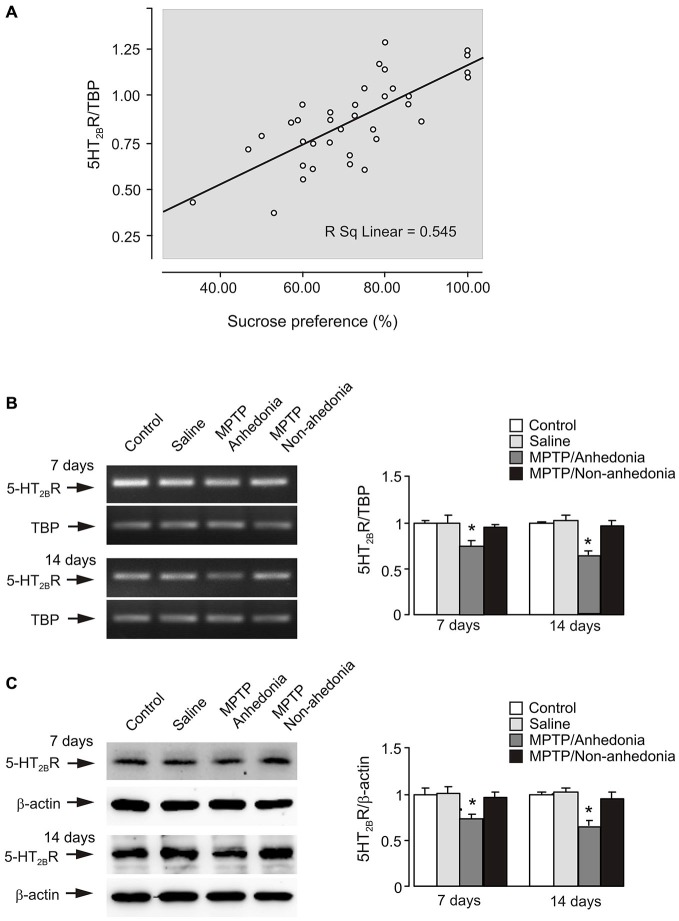
**5-HT_2B_ receptor expression in the brain of anhedonia mice. (A)** Correlation analysis between gene expression of 5-HT_2B_ receptor mRNA and the data of behavior test. 5-HT_2B_ receptor expression showed a positive correlation with sucrose consumption (*p* = 0.000, *r* = 0.727). *P*-values were two-tailed, and *p* < 0.01 was considered significant. **(B)** mRNA expression measured by RT-PCR of 5-HT_2B_ receptor in cerebral cortex* in vivo* from anhedonia or non-anhedonia mice. A representative experiment showing mRNA for 5-HT_2B_ receptor and for TBP, as a housekeeping gene. The size of PCR product of 5-HT_2B_ receptor is 370 bp, and that of TBP 236 bp. Similar results were obtained in three independent experiments. Average mRNA expression was quantified as ratios between 5-HT_2B_ receptor and the housekeeping TBP gene. Ratios between 5-HT_2B_ receptor and TBP in control group were designated a value of one. SEM values are indicated by vertical bars. *Indicates statistically significant (*p* < 0.05) difference from control and saline group at the same treatment period. **(C)** Protein level measured by immunoblotting of 5-HT_2B_ receptor (55 kDa) in cerebral cortex *in vivo* from anhedonia or non-anhedonia mice. Immunoblots from representative experiments. Bands of 55 and 42 kDa represent 5-HT_2B_ receptor and β-actin, respectively. Similar results were obtained in three independent experiments. Average protein level was quantified as ratios between 5-HT_2B_R and β-actin. Ratios between 5-HT_2B_ receptor and β-actin in control group were designated a value of one. SEM values are indicated by vertical bars. *Indicates statistically significant (*p* < 0.05) difference from other groups at the same treatment period.

Spearman correlation analysis was carried out to determine the relationship between gene expression of 5-HT_2B_ receptor mRNA and the data of behavior test. Scatter plots was made between data of sucrose consumption and gene expression of 5-HT_2B_ receptor mRNA (Figure 4A). From 3–21 days of MPTP treatment 5-HT_2B_ receptor expression correlates positive with sucrose consumption (*p* = 0.000, *r* = 0.727). This suggests that there is a positive relationship between the behavior change and receptor expression.

### 5-HT_2B_ Receptor Expression in Brains of Anhedonia Mice

Subsequently, we analyzed sucrose consumption in each MPTP-treated mouse with average value in control group by *t* test. Exposure to MPTP resulted in anhedonia in 33.3% of C57BL/6 mice (three out of nine animals) after 7 days treatment and 66.7% (6 out of 9 animals) after 14 days, while this percentage was 50% in CMS-treated CD-1 mice (Li et al., 2012). In non-anhedonia animals (*n* = 3) exposed to MPTP sucrose consumption was 75.19 ± 3.27% or 76.19 ± 1.19% after 7 or 14 days of treatment (*p* > 0.05) compared to control and saline groups), while in anhedonia animals (*n* = 6) it decreased to 57.65 ± 2.35% or 51.61 ± 4.40% (*p* < 0.05 compared to control and saline groups) after 7 or 14 days, respectively. Figures 4B,C shows mRNA and protein expression of 5-HT_2B_ receptor in control, saline, anhedonia and non-anhedonia groups after 7 and 14 days of treatment. Gene expression of 5-HT_2B_ receptor was significantly decreased in anhedonia group but not in non-anhedonia group.

### Effects of Fluoxetine on Gene Expression of 5-HT_2B_ Receptor and Behavior Changes of Anhedonia Mice

**Figure 5:**
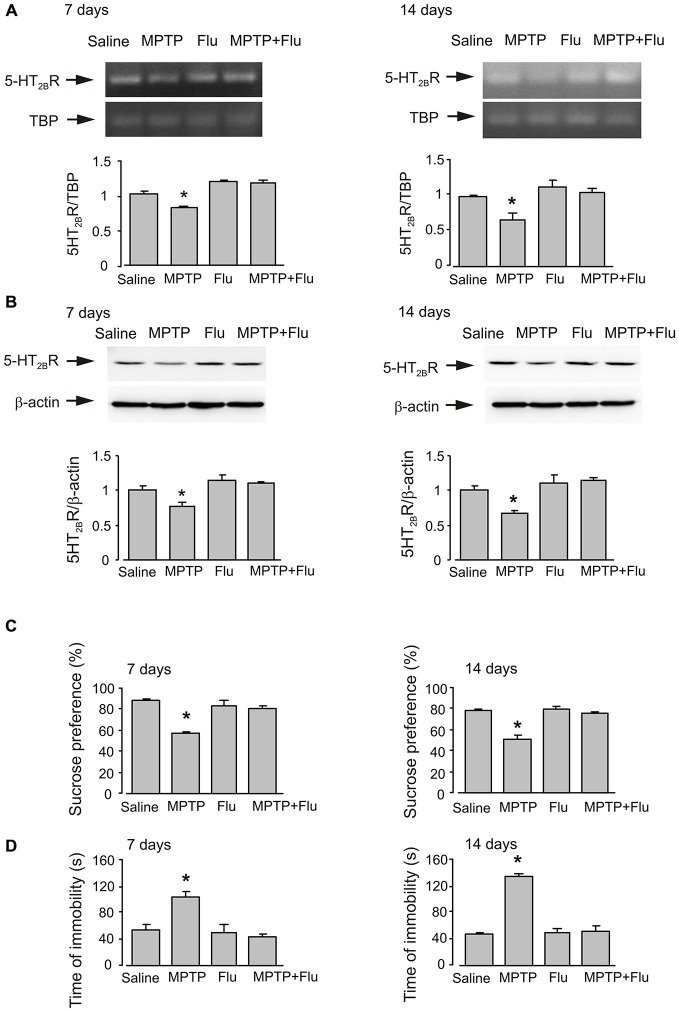
**Effects of fluoxetine on gene expression of 5-HT_2B_ receptor and behavior changes of anhedonia mice**. After either 7 days or 14 days (7 days after the end of MPTP treatment) of MPTP treatment, mice with anhedonia were daily injected intraperitoneally with fluoxetine (10 mg/kg/d dissolved in 0.9% NaCl) or saline for 14 days. **(A)** mRNA expression of 5-HT_2B_ receptor was measured by RT-PCR. A representative experiment showing mRNA for 5-HT_2B_ receptor and for TBP, as a housekeeping gene. The size of PCR product of 5-HT_2B_ receptor is 370 bp, and that of TBP 236 bp. Similar results were obtained in three independent experiments. Average mRNA expression was quantified as ratios between 5-HT_2B_ receptor and the housekeeping TBP gene. Ratios between 5-HT_2B_ receptor and TBP in control group were designated a value of one. SEM values are indicated by vertical bars. *Indicates statistically significant (*p* < 0.05) difference from other groups. **(B)** Protein level measured by immunoblotting of 5-HT_2B_ receptor (55 kDa). Immunoblots from representative experiments. Bands of 55 and 42 kDa represent 5-HT_2B_ receptor and β-actin, respectively. Similar results were obtained in three independent experiments. Average protein level was quantified as ratios between 5-HT_2B_R and β-actin. Ratios between 5-HT_2B_ receptor and β-actin in control group were designated a value of one. SEM values are indicated by vertical bars. *Indicates statistically significant (*p* < 0.05) difference from other groups. **(C)** Percentage of sucrose preference. Values were expressed as the mean ± SEM (7 days, *n* = 5; 14 days, saline: *n* = 6, MPTP: *n* = 6, flu: *n* = 5, MPTP plus flu: *n* = 6). *Indicates statistically significant (*p* < 0.05) difference from other groups at the same treatment period. **(D)** Forced-swimming test. The values of immobility time are expressed as the mean ± SEM (7 days, *n* = 5; 14 days, saline: *n* = 6, MPTP: *n* = 6, flu: *n* = 5, MPTP plus flu: *n* = 6). *Indicates statistically significant (*p* < 0.05) difference from other groups at the same treatment period.

After either 7 days or 14 days (7 days after the end of MPTP treatment) of MPTP treatment, mice with anhedonia were daily injected intraperitoneally with fluoxetine (10 mg/kg/d dissolved in 0.9% NaCl) or saline for 14 days. Exposure to MPTP for 7 days decreased mRNA and protein expression to 80.3 and 77.3% of control levels, respectively (*n* = 6, *p* < 0.05; Figures 5A,B). It was further decreased to 66.8 and 67.4% after 14 days of MPTP treatment (*n* = 6, *p* < 0.05). Chronic treatment with fluoxetine up-regulated gene expression of 5-HT_2B_ receptor to its normal levels at both time points (*n* = 6, *p* < 0.05).

Fluoxetine corrected MPTP-induced decrease of sucrose consumption (Figure 5C) and time of immobility in forced-swimming test (Figure 5D)

### Effects of MPTP on Motor Activity

**Figure 6:**
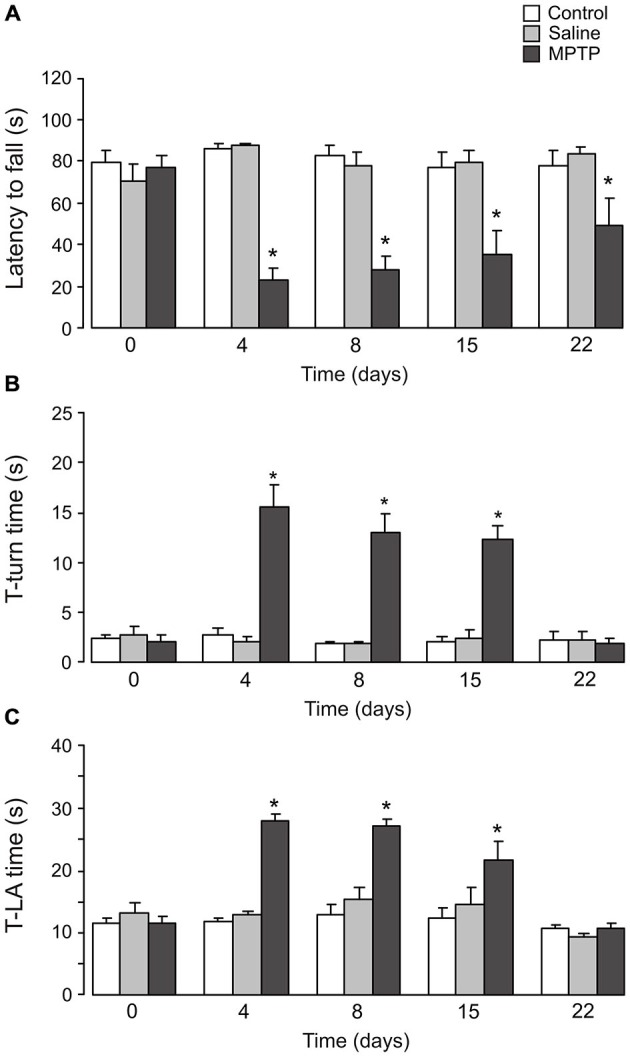
**Effects of MPTP on motor activity**. C57BL/6 mice were treated with MPTP (25 mg/kg/d, 7 days, i.p.). Test was done on the 3rd day, 7th day (1 day after the cease of MPTP treatment), 14th day (7 days after the cease of MPTP treatment) and 21th day (14 days after the cease of MPTP treatment). **(A)** The rotarod test. The latency to fall off the rotarod was recorded. The values are expressed as the mean ± SEM, *n* = 6, *Indicates statistically significant (*p* < 0.05) difference from other groups at the same treatment period. **(B)** T-turn time. The time taken for the mice to turn completely downward in the pole test. The values are expressed as the mean ± SEM, *n* = 6, *Indicates statistically significant (*p* < 0.05) difference from other groups at the same treatment period. **(C)** T-LA time. The time taken for the mice to reach the floor in the pole test. The values are expressed as the mean ± SEM, *n* = 6, *Indicates statistically significant (*p* < 0.05) difference from other groups at the same treatment period.

On rotarod performance test, the latency of fall (Figure 6A) in mice treated with MPTP was significantly shorter than in control groups after 3 days (control: 86.3 ± 2.4 s; saline: 87.3 ± 1.6 s; MPTP: 22.7 ± 5.4 s; *n* = 6, *p* < 0.05) and 7 days (control: 82.8 ± 4.4 s; saline: 78.4 ± 5.9 s; MPTP: 27.8 ± 6.1 s; *n* = 6, *p* < 0.05) of MPTP treatment. Although the animals could stay on the rotating rod longer after 14 days (7 days after the end of MPTP treatment; control: 77.2 ± 7.1 s; saline: 79.3 ± 6.3 s; MPTP: 34.9 ± 12.0 s; *n* = 6, *p* < 0.05) and 21 days (14 days after the cease of MPTP treatment; control: 77.7 ± 7.5 s; saline: 83.7 ± 3.1 s; MPTP: 48.5 ± 13.7 s; *n* = 6, *p* < 0.05), the latencies of fall in these mice were still significantly shorter than control groups.

Treatment with MTPT also impaired performance in the pole test. The time taken by the mice to turn completely downward (Figure 6B; T-turn time) was significantly increased after 3 days (control: 2.8 ± 0.7 s; saline: 2.1 ± 0.4 s; MPTP: 15.7 ± 2.1 s; *n* = 6, *p* < 0.05), 7 days (control: 1.9 ± 0.2 s; saline: 1.9 ± 0.3 s; MPTP: 13.1 ± 1.8 s; *n* = 6, *p* < 0.05) and 14 days (7 days after the end of MPTP treatment; control: 2.1 ± 0.5 s; saline: 2.5 ± 0.9 s; MPTP: 12.3 ± 1.5 s; *n* = 6, *p* < 0.05) of the experiment. However, after 21 days of experiment the pole test performance showed no difference between control and MTPT-treated mice (control: 2.3 ± 0.9 s; saline: 2.3 ± 0.8 s; MPTP: 2.0 ± 0.5 s; *n* = 6, *p* > 0.05). Similar results were also observed in the time taken to reach the floor (Figure 6C; T-LA time).

### Effects of MPTP on Sickness Behaviors

Effect of MPTP on sickness behaviors is shown in Table 1. It includes body weight, lines crossed and social exploration(s). Only body weight showed significant decrease at 7 days (saline: −0.75 ± 0.62%, *n* = 15; MPTP: −5.95 ± 1.07%, *n* = 26; *p* < 0.05) and 14 days (7 days after the end of MPTP treatment; saline: −0.64 ± 1.39%, *n* = 15; MPTP: −5.25 ± 0.99%, *n* = 22; *p* < 0.05). MPTP had no effect on lines crossed and social exploration(s) at all the time points examined.

**Table 1:** **Effect of MPTP on sickness behaviors (body weight changes, locomotor activity and social exploratory behavior)**.

		0 Day	3 Days	7 Days	14 Days	21 Days
Body weight changes (%)	Saline	–	−1.11 ± 1.38	−0.75 ± 0.62	−0.64 ± 1.39	−0.67 ± 1.28
			(*n* = 15)	(*n* = 15)	(*n* = 15)	(*n* = 18)
	MPTP	–	−4.30 ± 1.02	−5.95 ± 1.07*	−5.25 ± 0.99*	−0.05 ± 1.18
			(*n* = 13)	(*n* = 25)	(*n* = 22)	(*n* = 14)
Lines crossed	Saline	57.17 ± 5.18	51.50 ± 8.84	54.33 ± 7.80	57.50 ± 6.30	50.00 ± 6.13
		(*n* = 6)	(*n* = 6)	(*n* = 6)	(*n* = 6)	(*n* = 6)
	MPTP	47.00 ± 7.00	49.12 ± 5.98	52.71 ± 5.44	55.86 ± 4.91	50.86 ± 4.80
		(*n* = 7)	(*n* = 7)	(*n* = 7)	(*n* = 7)	(*n* = 7)
Social exploration (s)	Saline	147.00 ± 18.15	146.60 ± 11.27	150.00 ± 23.00	152.20 ± 8.29	171.60 ± 13.19
		(*n* = 5)	(*n* = 5)	(*n* = 5)	(*n* = 5)	(*n* = 5)
	MPTP	151.20 ± 13.38	150.67 ± 14.95	161.83 ± 18.16	139.67 ± 12.83	152.33 ± 6.56
		(*n* = 5)	(*n* = 6)	(*n* = 6)	(*n* = 6)	(*n* = 6)

*Significantly different from the corresponding saline group (*p* < 0.05).

### Effect of MPTP on TH Expression in Dopaminergic Neurons

**Figure 7:**
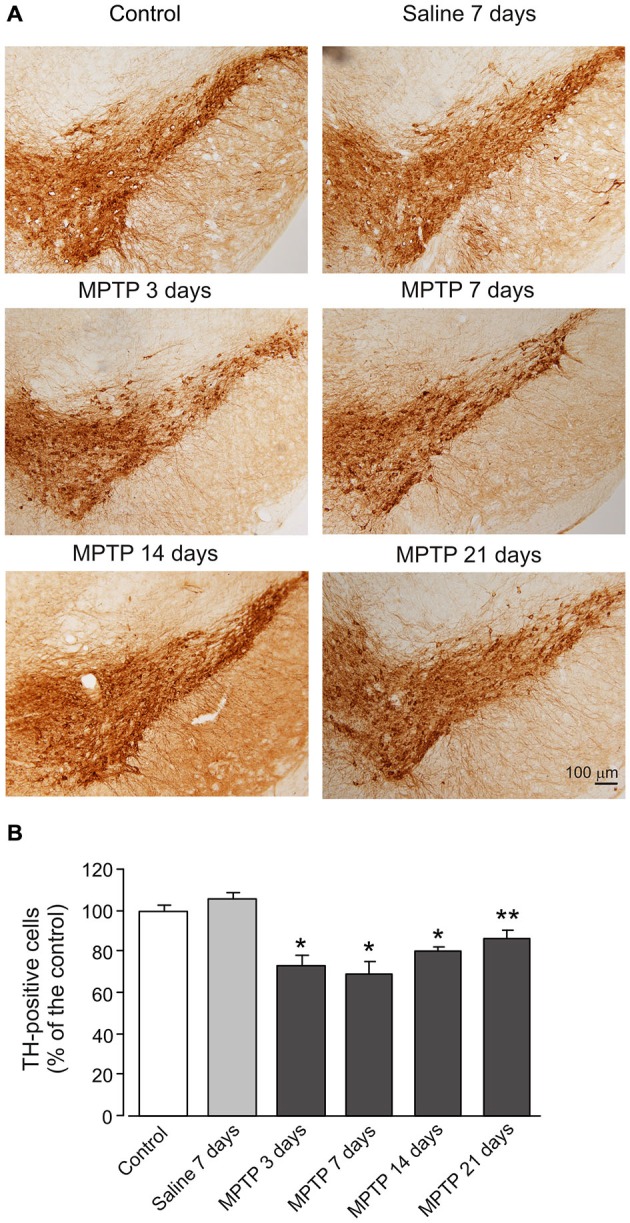
**MPTP induced reduction of TH expression in dopaminergic neurons. (A)** Photomicrographs of representative SN sections stained with an antibody against TH. Scale bar = 100 μM. **(B)** Quantitative analysis of TH-positive neuron numbers in the SN. The values are expressed as the mean ± SEM, *n* = 7. *Indicates statistically significant (*p* < 0.05) difference from control and saline groups. **Indicates statistically significant (*p* < 0.05) difference between control, saline and MPTP 7 days groups.

Treatment with MPTP significantly decreased number of TH-positive cells in sections of SN (Figure 7). The number of TH cells begun to decrease after 3 days of treatment (73.3 ± 5.35% of control group, *n* = 7, *p* < 0.05). After 7 days of treatment the decrease in cell number reached its maximum (68.7 ± 6.44% of control group, *n* = 7, *p* < 0.05). After 14 and 21 days treatment, the cell number began to recover but remained significantly lower than in control (14 days: 80.0 ± 2.75% of control group, *n* = 7, *p* < 0.05; 21 days: 86.6 ± 3.69% of control group, *n* = 7, *p* < 0.05).

## Discussion

Depression is one of the most common non-motors symptoms in PD, with prevalence above 50% (Pachana et al., 2013). The pathophysiological mechanisms underlying depression in PD are far from being clear; possibly these are related to aberrations in serotonergic, adrenergic or dopaminergic neurotransmission (Ossowska and Lorenc-Koci, 2013). In clinical context, depression in PD, when compared to major depression, exhibits high degree of dysphoria, irritability, and pessimism about the future, with lower incidence of inadequacy, senses of guilt, sorrow and shame (Lieberman, 2006). Incidentally, dopaminergic agonists demonstrate antidepressant effect in PD patients and the recovery rate is better than after treatment with classical antidepressant drugs (Ossowska and Lorenc-Koci, 2013, and references therein). Rats systemically injected with MPTP for 18 days (Kryzhanovskiı et al., 1995) as well as in rats which received MPTP injection into substantia nigra (Santiago et al., 2010) developed anhedonia (as measured by sucrose consumption) very similarly to our results presented in this paper.

To the best of our knowledge, our present work is the first that suggests the role of astroglial 5-HT_2B_ receptors in the pathophysiology of depression associated with MPTP model of PD. The 5-HT_2B_ receptors, discovered in Foguet et al. (1992), and classified the 5-HT_2B_ receptors in Schmuck et al. (1994) are expressed in various types of brain cells. First, they were found in Purkinje neurones (Choi and Maroteaux, 1996) and later the specific 5-HT_2B_ receptor mRNAs was identified in freshly isolated fractions of neurons and astrocytes from adult mice (Li et al., 2012) in which cell-specific markers were linked with differently fluorescent compounds thus allowing cell separation using FACS. The relevance of 5-HT_2B_ receptors to depression is corroborated by (i) the decrease of its gene expression in the nervous tissue of animals developing anhedonia under the CMS, in non-anhedonic animals that were subjected to the same CMS protocol expression of 5-HT_2B_ receptors is unchanged (Li et al., 2012); (ii) the up-regulation of 5-HT_2B_ receptor gene expression by chronic treatment with fluoxetine in astrocytes in cultures and freshly isolated from the *in vivo* brains (Li et al., 2009, 2012); and (iii) the dependence of antidepressant effect of SSRIs on 5-HT_2B_ receptors *in vivo* (Diaz et al., 2012). Our present study shows that the down-regulation of gene expression of 5-HT_2B_ receptors occurs specifically in astrocytes in parallel with the development of depressive behaviors in MPTP animal model of PD. In addition, fluoxetine up-regulated expression of 5-HT_2B_ receptors gene in the brains of MPTP-treated animals to the level of control group and corrected the depression behaviors-induced by MPTP. This finding provides another evidence that astrocytic 5-HT_2B_ receptor is involved in depression.

Sickness behavior is a coordinated set of adaptive behavioral changes, including depression that develop in the course of an infection. It has been suggested that sickness behavior is general responses to systemic inflammation, resulting from peripheral stimulus-induced production of cytokines in the brain (Ferrari and Tarelli, 2011), which may complicate in the pathophysiology of PD. In the present study, we found that MPTP treatment significantly reduced body weight. We cannot exclude the involvement of sickness behavior in MPTP model since in mice MPTP treatment indeed stimulated plasma IL-1 and IL-6 (Luo et al., 2004). However, there is no evidence that fluoxetine effects on sickness behavior or 5-HT_2B_ receptors are reduced by systemic inflammatory and brain cytokines.

The mechanism(s) underlying the down-regulation of astroglial 5-HT_2B_ receptors upon MPTP treatment is unknown, although its development shares the time course of extinction of TH-positive neurons in the striatum. The down-regulation of serotonin receptors in astrocytes may also reflect global deficit of serotonergic system in PD. The levo-dopa is known to potentiate serotonergic system, which is believed to be responsible for levo-dopa-induced diskenisia, whereas sarizotan (EMD128130), an agonist of 5-HT_1A_ receptor, is used in clinic because of its inhibitory effect on 5-HT release (Bibbiani et al., 2001). The MPTP also seems to be toxic for cortical serotonin axons (Nayyar et al., 2009), which could directly affect serotonergic transmission. In the brains of PD patients and of MTPT-treated primates concentration of 5-HT in the prefrontal cortex is decreased (Scatton et al., 1983; Pérez-Otaño et al., 1991; Pifl et al., 1991; Russ et al., 1991). In the mouse brain, MPTP effect on 5-HT system is not consistent, probably reflecting the differences in experimental protocols. In the acute treatment, i.e., 4 intra-peritoneal injections of MPTP (20 mg/kg) over an 8 h period significantly decreased dopamine level in striatum (Nayyar et al., 2009; Gorton et al., 2010; De Benedetto et al., 2014), whereas 5-HT concentration was decreased in the somatosensory and medial prefrontal cortex, and the density of serotonergic axons was decreased in the medial prefrontal cortex (Nayyar et al., 2009). In rats which received direct injection of MPTP into substantia nigra, hippocampal reduction in 5-HT concentration was accompanied with depression-like behaviors (Santiago et al., 2010). However, in sub-acute treatment, similar to that used in the present study, MPTP did not induce any significant changes in 5-HT concentration in the brain (Nayyar et al., 2009). Nevertheless, we have to keep in mind that in all the studies mentioned above, 5-HT concentrations were measured as total concentration in the brain homogenates and not extracellular transmitter released from neural cells. In addition, MPTP also decreased 5-HT_2B_ receptors expression in cultured astrocytes, suggesting that this phenomenon may be only applicable to MPTP induced Parkinson’s conditions. Although this hypotheses can be tested in other animal models, such as 6-hydroxydopamine (6-OHDA), this compound also has direct effect on astrocytes (Zhang et al., 2005). We probably cannot make final conclusion without postmortem examination of 5-HT_2B_ receptors in patients’ brain.

We have studied astrocytic 5-HT_2B_ receptor extensively in recent years. In astrocytes 5-HT has much higher affinity to 5-HT_2B_ receptor than to 5-HT_2C_ receptor (Li et al., 2010). Various SSRIs activate astroglial 5-HT_2B_ receptors and induce EGFR transactivation (Zhang et al., 2010), which, in turn, regulates expression of multiple genes (for review, see Peng and Huang, 2012; Hertz et al., 2015). Among these genes, expression of 5-HT_2B_ receptor and cPLA_2_ are significantly reduced in mice that become anhedonic after chronic stress, but not in those that does not develop anhedonia (Li et al., 2012). We have demonstrated recently that chronic treatment with fluoxetine up-regulates these two genes in astrocytes in the brains of anhedonic animals (Dong et al., 2015). This is in agreement with our present findings that astrocytic 5-HT_2B_ receptor is decreased in mice that become anhedonic, but not in those that does not develop anhedonia in MPTP-treated mice. Our study suggests that astrocytic 5-HT_2B_ receptor may be also involved in depression associated with the PD and up-regulation of astrocytic 5-HT_2B_ receptor expression by chronic treatment with fluoxetine may be one the mechanism of action of antidepressants.

## Author Contributions

YR, LP conceived and designed experiments; XZ, DS and LG collected and analyzed the data; LP and AV interpreted the data and wrote the paper. All authors commented on the manuscript and have approved the final version.

## Conflict of Interest Statement

The authors declare that the research was conducted in the absence of any commercial or financial relationships that could be construed as a potential conflict of interest.
